# Static elastic cloaking, low-frequency elastic wave transparency and neutral inclusions

**DOI:** 10.1098/rspa.2019.0725

**Published:** 2020-08-05

**Authors:** Andrew N. Norris, William J. Parnell

**Affiliations:** 1 Department of Mechanical and Aerospace Engineering, Rutgers University, Piscataway, NJ 08854-8058, USA; 2 Department of Mathematics, University of Manchester, Oxford Road, Manchester M13 9PL, UK

**Keywords:** elastic cloaking, elastic wave scattering, neutral inclusions

## Abstract

New connections between static elastic cloaking, low-frequency elastic wave scattering and neutral inclusions (NIs) are established in the context of two-dimensional elasticity. A cylindrical core surrounded by a cylindrical shell is embedded in a uniform elastic matrix. Given the core and matrix properties, we answer the questions of how to select the shell material such that (i) it acts as a static elastic cloak, and (ii) it eliminates low-frequency scattering of incident elastic waves. It is shown that static cloaking (i) requires an anisotropic shell, whereas scattering reduction (ii) can be satisfied more simply with isotropic materials. Implicit solutions for the shell material are obtained by considering the core–shell composite cylinder as a neutral elastic inclusion. Two types of NI are distinguished, *weak* and *strong* with the former equivalent to low-frequency transparency and the classical Christensen and Lo generalized self-consistent result for in-plane shear from 1979. Our introduction of the *strong NI* is an important extension of this result in that we show that standard anisotropic shells can act as perfect static cloaks, contrasting previous work that has employed ‘unphysical’ materials. The relationships between low-frequency transparency, static cloaking and NIs provide the material designer with options for achieving elastic cloaking in the quasi-static limit.

## 1. Introduction

The ability to cloak a region of space so that an incident field or applied loading does not see or feel the presence of an object is of great interest in science and engineering. Over the last two decades, significant progress has been made in this field in the domains of electromagnetism [1,2], acoustics [3,4], heat transport [5] and flexural waves on thin plates [6,7]. Cloaking of elastic waves however, even in the quasi-static limit requires materials with properties that are, at present, unachievable. According to transformation elasticity [8,9], one needs solids that display a significant amount of anisotropy combined with strong asymmetry of the elastic stress. Large anisotropy is common in composite materials and can be engineered by design, but significant stress asymmetry is not seen in practical materials. Some mechanisms to circumvent apparent non-feasibility of cloaking in elastodynamics have been proposed, including isotropic polar solids for conformal transformation elasticity and cloaking [10,11], hyperelastic materials under pre-stress [12,13], symmetrization of the asymmetric effective modulus tensor [14], and under some circumstances solutions can be found in the case of thin plates that do not need asymmetric stress [15,16]. Recent work has employed lattice transformations to cloak in-plane shear waves [17].

Restricting attention to statics on the other hand, a purely static cloak is an elastic layer that has the effect of ensuring that the deformation exterior to the cloaked region is the same as if there were no object or layer present. Static cloaking is closely related with the concept of a *neutral inclusion* (NI), which is a region of inhomogeneity in an otherwise uniform solid that does not disturb an applied exterior field. NIs can be tailored to specific loading types, whereas a static cloak will ensure that there is no influence to the presence of an object for *any* type of imposed field. NIs are therefore by definition statically cloaked for a certain imposed field. Examples of NIs are Hashin’s coated sphere [18] for conductivity, later generalized to coated confocal ellipsoids [19] and other possible shapes [20]. The associated scalar potential problem and associated NIs and coated NIs have been studied extensively, see [21, §7], [22] and references therein. Extensions to the case of nonlinear conductivity [23] and hydrostatic loading in plane finite elasticity have also been considered [24]. The two-dimensional scalar potential problem is pertinent in the context of the anti-plane elastic problem [20,25]. The full elastostatic NI problem is more challenging, and there have been a number of relevant studies in linear elasticity [22,26–31]. A general elastic NI, or a condition to realize one, has not been exhibited with finite thickness shells, although see [22] where NIs are derived for special loading scenarios. Instead it is often the case that ‘interface’-type conditions are required for the combined shear/bulk modulus neutrality, i.e. for neutrality to be achieved under general in-plane loadings [27]. There has been some success in realization of an approximate core–shell design based on Hashin’s assemblage using a pentamode material for the shell, a so-called unfeelability cloak [32].

**Figure 1.**
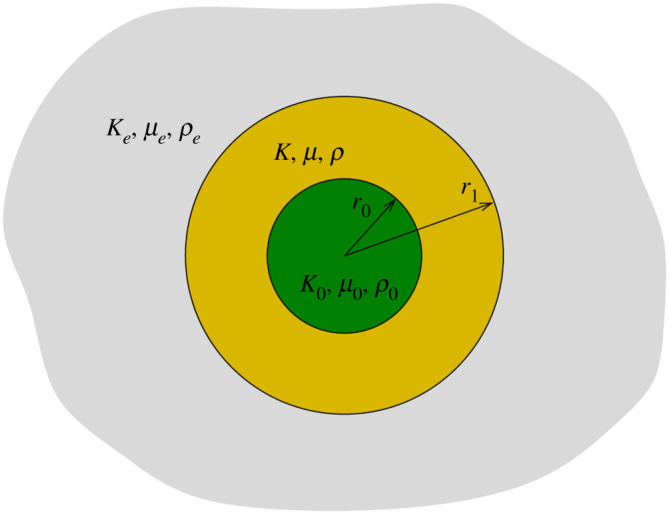
The central cylindrical core of radius *r*_0_ is surrounded by a cylindrical shell (or layer or annulus) of elastic material of outer radius *r*_1_. The core density, shear modulus and in-plane bulk modulus are *ρ*_0_, *μ*_0_ and *K*_0_, the shell properties can be anisotropic and radially dependent, although only the isotropic shell case is illustrated here. The composite cylinder lies in an infinite uniform isotropic elastic medium, *ρ*_*e*_, *μ*_*e*_ and *K*_*e*_. (Online version in colour.)

Our interest here is two-dimensional cylindrical and inhomogeneous NIs for elasticity. The configuration studied is a cylindrical core region surrounded by a shell (or coating, layer, annulus) all of which is embedded in a host exterior medium, as illustrated in figure 1. The properties of the shell (homogeneous or inhomogeneous) are chosen so that the combined core and coating act as an NI. Unlike the cloak of transformation elastodynamics [9], the moduli of the static cloak will depend upon the properties of the cloaked object. The three-phase elastically isotropic configuration depicted in figure 1 has been studied previously, under the action of various far-field loadings in the context of estimating the static effective properties of core unidirectional fibres dispersed in a matrix with the properties of the coating. By inserting the composite cylinder (core plus coating) in the background medium and requiring that it act as an NI, the background properties provide a *self-consistent* estimate of the effective material properties, i.e. the matrix properties. This approach partitions into two sub-problems, first for in-plane hydrostatic loading which ensures a condition can be determined for the effective bulk modulus. However the in-plane shear problem is under-determined; the ‘perturbed’ static displacement *outside* the composite cylinder depends on two coefficients, while the background material has only a single parameter: the effective shear modulus. It is not possible to make both perturbed displacement coefficients vanish simultaneously, i.e. the composite cylinder cannot be a NI/static cloak *if the coating is isotropic*. As an alternative, Christensen & Lo [33] assumed that the strain energy in the composite cylinder must be the same as the strain energy in an equivalent volume of the effective material, which can be satisfied by setting only one of the displacement coefficients to zero (the stress terms associated with 1/*r*^4^ decay do not contribute to the strain energy). This procedure has been termed the Generalized Self Consistent Scheme (GSCS) [34]. The GSCS energy equivalence method has been generalized to the case of multiply layered cylinders using transfer matrices [35]. The effect of anisotropy in fibres and coatings was considered by Avery & Herakovich [36] in relation to thermal properties of composites. Thermoelastic effective properties for orthotropic phases were derived using a combination of the GSCS and Composite Cylinders Assemblage (CCA) methods in [34]. A Mori–Tanaka inspired interaction approach was used by Chen *et al*. [37] to consider thermomechanical loading of cylindrically orthotropic fibres with transversely isotropic coatings. The solutions were subsequently applied to estimate effective properties of coated cylindrically orthotropic fibre reinforced composites [38]. Recent reviews of relevance include [39] on homogenization and micromechanics and [40,41] on inclusions.

Christensen and Lo’s solution for the composite cylinders model [33], and its generalizations [34,35] can be considered as *weak NIs* because the perturbed exterior field is not completely eliminated (the 1/*r*^4^ decay in the exterior stress remains) as compared with *strong NIs* for which the exterior displacement and stress are unperturbed. A related but apparently quite distinct situation arises with scattering of time-harmonic elastic waves. The scattered, i.e. perturbed, exterior field, can be expressed as an asymptotic series in a non-dimensional parameter proportional to the frequency. We say that the scattering object is *transparent at low frequency* if both the leading-order longitudinal and transverse-scattered waves vanish [42]. The lowest-order terms in the power series vanish for both the scattered longitudinal and transverse waves if the scatterer is an NI. However, as discussed above, a given two phase composite cylinder with isotropic phases can at most be a weak NI, which begs the question of how the weak NI relates to low-frequency transparency.

One lesson taken from the composite cylinders model [33] is that isotropy of the shell is not sufficient for the composite cylinder to act as a strong NI. We therefore anticipate that anisotropy is required. One of our main results is therefore to identify the type of anisotropy necessary to achieve a strong NI effect. The static elastic cloak determined here is however distinct from others in elasticity [8–13] in that it can be realized using ‘normal’ elastic materials corresponding to symmetric stress.

### (a) Objective and overview

The problem considered is as follows: given a host matrix and a cylindrical core, determine the shell properties such that the core+shell (composite assemblage) acts as either a strong or a weak NI, to planar deformation of hydrostatic and shear loading (figure 1). We define the following terms, both of which apply under homogeneous loading states.

— *Strong NI*: the perturbed field exterior to the NI is zero. This is equivalent to a static elastic cloak.
— *Weak NI*: the strain energy of the NI is the same as the strain energy of an equivalent volume of matrix material.

The core and matrix have material properties *K*_0_, *μ*_0_, *ρ*_0_ and *K*_*e*_, *μ*_*e*_, *ρ*_*e*_, respectively, the *in-plane* bulk modulus, shear modulus and density. Given the core radius *r*_0_ and the outer radius of the shell *r*_1_, the objective is to find properties of the shell that result in an NI of either type. At the same time, we are interested in the relation between NI effects, weak and strong, and low-frequency transparency.

We will explicitly show that a two-phase composite cylinder with isotropic core and shell cannot be a strong NI. An isotropic shell can only yield a weak NI, and equations for the required properties *K*, *μ*, *ρ* will be obtained. A strong NI requires that the shell be anisotropic, and the requisite conditions will be found. It will be shown that the NI and transparency properties are related: a weak NI is transparent at low frequency, that is, both of the leading-order scattered waves (longitudinal and transverse) vanish. Conversely, low-frequency transparency implies that the scatterer acts as an NI, weak or strong, but generally weak.

Given a composite cylinder and its properties one can ask what are the properties of the matrix which makes it act as an NI. This is a standard *effective medium* problem, which may be solved in an approximate or exact manner, as we will see in §§2 and 5, respectively. Finding solutions for the NI layer properties is therefore an inverse problem: we will first solve the effective medium problem, with the NI properties determined as implicit functions of the core and matrix properties. The approximate effective medium solution in §2 provides the only explicit examples for the NI properties.

Our approach to the exact solution of the NI problem combines static and dynamic solutions in a novel manner. Unlike previous derivations of the effective bulk modulus, which require full solutions for the displacement and stress fields in the composite cylinder [33,35], here it is found directly as the solution of an ordinary differential equation (ODE) of Riccati type. The effective shear modulus involves a 2 × 2 impedance matrix which satisfies a Riccati ODE. This matrix yields both the low-frequency transparency and the NI conditions. The former is derived using a low-frequency expansion of the scattered field, giving a condition identical to the GSCS. The NI condition for shear is a purely static one which reduces to a single constraint on the elements of the impedance matrix. In particular, we derive a simple condition which is necessary and sufficient to obtain a strong NI. We provide examples of composite cylinders comprising isotropic cores and uniform anisotropic shells that are strong NIs and illustrate two of these cases graphically showing the difference between the weak and strong NI in the process.

The outline of the paper is as follows. An approximate effective medium solution is used in §2 to solve for (approximate) NI parameters. The explicit solution shows that the range of possibilities decreases to zero in certain parameter regimes. Section 3 outlines the exact forward solution approach for the composite cylinder effective medium problem, and relates the NI effect to low-frequency transparency effects. By representing the fields in terms of angular harmonics, it is apparent that there are two distinct problems to solve: for *n* = 0 and *n* = 2. Solutions of the effective medium problem are given in §4 for the effective bulk modulus (*n* = 0), and in §5 for the effective shear modulus (*n* = 2). Distinction between weak NI, strong NI and low-frequency transparency become apparent in §5, where the exact NI solution is described. Examples of strong NI core–shell composite cylinders are presented in §6. Concluding remarks are given in §7.

## 2. Static cloak using an approximate model

As an introduction to the problem, we first demonstrate how one can use an *approximate* model to estimate the properties necessary for an approximate static cloak. It should be stressed that since the model is approximate the configuration cannot be classified as an exact NI of any type, weak or strong. However, it gives an indication of what is required of such an NI and its possible regimes of validity. Consider the single-core configuration as depicted in figure 1. In the context of effective medium theory, the core (subscript 0) and coating properties, together with core volume fraction *f* ∈ (0, 1), are given and the external properties (subscript *e*) are then determined subject to some consistency constraint. The static cloak problem is different in that the core and surrounding medium properties are given and the cloak (coating) properties are chosen in order to render either equivalent energy or zero transparency, etc.

As an example, let us use effective property estimates based on a modification of the Kuster–Toksöz model [43, eqn (4)]

2.1a $$\begin{array}{rl} & \rho -\rho_{e}=f(\rho -\rho_{0}), \end{array}$$

2.1b $$\begin{array}{rl} & \frac{K-K_{e}}{\mu +K_{e}}=f\;\left(\frac{K-K_{0}}{\mu +K_{0}}\right), \end{array}$$

2.1c  μ−μeμ+μe(1+2μK00)=f(μ−μ0)μ+μ0(1+2μK00)

where $f=r_{0}^{2}/r_{1}^{2}$. The relation (2.1a) for densities is obviously correct and therefore we will not consider density further. The expression (2.1b) is, as we will see, the correct relation between *K*, *K*_*e*_, *K*_0_ and *μ* for isotropic shells. However, the shell shear modulus *μ* given by (2.1c) is not the right value, but an approximation. The identities (2.1b,*c*) coincide with the Hashin–Shtrikman two-dimensional bounds for *K*_*e*_ and *μ*_*e*_ [44], similar to the three-dimensional Kuster–Toksöz model [45]; formulae valid in the limit of small *f* were derived in [46, eqns (3.15), (3.16)] which are in agreement with (2.1c). Solving for the layer or cloak properties yields

2.2a $$\rho =\frac{\rho_{e}-f\rho_{0}}{1-f}$$

and

2.2b $$K=\frac{K_{e}/(\mu +K_{e})-fK_{0}/(\mu +K_{0})}{1/(\mu +K_{e})-f/(\mu +K_{0})},$$

where *μ* solves the cubic equation

2.2c $$\begin{array}{rl} & [(1+f)(\mu_{0}-\mu_{e})K_{e}K_{0}-(K_{e}+2K_{0}-f(2K_{e}+K_{0}))\mu_{e}\mu_{0}]\;\mu \\ & \;+[(\mu_{0}-\mu_{e})(K_{e}+fK_{0})+(1-f)(K_{e}K_{0}-2\mu_{e}\mu_{0})+2\mu_{0}K_{0}-f2\mu_{e}K_{e}]\;\mu^{2}\\ & \;+[K_{e}+2\mu_{0}-f(K_{0}+2\mu_{e})]\;\mu^{3}-(1-f)\mu_{0}\mu_{e}K_{0}K_{e}=0. \end{array}$$

A solution exists for (*ρ*, *K*, *μ*) for any given (*ρ*_0_, *K*_0_, *μ*_0_), (*ρ*_*e*_, *K*_*e*_, *μ*_*e*_) if *f* is small. As *f* is increased, the solution may or may not exist. If *ρ*_0_ > *ρ*_*e*_, then a positive solution for *ρ* is only possible for *f* < *ρ*_*e*_/*ρ*_0_. A small cloak is equivalent to large *f*, i.e. 1 − *f* ≪ 1.

For instance, in the limiting cases when the core is a hole, equation (2.2b,*c*) give

2.3 $$K=\frac{(1+2f)\mu_{e}}{1-2\nu_{e}(1+f))},\;\mu =\frac{(1+2f)\mu_{e}}{1-2f(1-2\nu_{e})},\;\text{for}\;K_{0}=\mu_{0}=0,$$

where $\nu_{e}=\frac{1}{2}-(\mu_{e}/2K_{e})$. Note that because the planar problem is isotropic, *ν*_*e*_ is given by the expression for the isotropic Poisson’s ratio in terms of in-plane properties *K* and *μ*, but since the effective medium is transversely isotropic it cannot be thought of as Poisson’s ratio: see the erratum follow up [47] to [33]. If the core is a rigid inclusion the cloaking layer becomes

2.4 $$\begin{array}{rl} & \mu =\frac{(2-f)\mu_{e}-(1+f)K_{e}}{2(2+f)}+\sqrt{{\left(\frac{(2-f)\mu_{e}-(1+f)K_{e}}{2(2+f)}\right)}^{2}+\left(\frac{1-f}{2+f}\right)K_{e}\mu_{e}},\\ & K=(1-f)K_{e}-f\mu ,\;\text{for}\;\frac{1}{K_{0}}=\frac{1}{\mu_{0}}=0. \end{array}$$

In each case, the cloak depends on the matrix properties and the core volume fraction *f*. The expression for *K* in (2.3), which must be positive and finite, implies that the range of possible *f* shrinks to zero as *ν*_*e*_ approaches 1/2, the incompressibility limit.

Solutions for static cloak properties (or NIs) are now sought that do not require approximate effective property expressions.

## 3. Quasi-static cloaking problem set-up

The objective is to determine necessary and sufficient conditions on the material properties of the coating of figure 1 in order that the combined core and coating acts as a quasi-static cloaking device. Two distinct definitions of quasi-static cloaking are considered: (i) the NI effect, and (ii) low-frequency wave transparency. The former is a purely static concept whereby an applied static field is unperturbed in the exterior of the core–shell composite. Low-frequency wave transparency is a dynamic concept; it requires that the leading-order term in the expansion of the scattered field expressed as an expansion in frequency vanishes for any type of incident time harmonic plane wave. However, as one might expect, it is possible to rephrase the condition in terms of static quantities, as in Rayleigh scattering [48]. This idea is used here also, and in the process the similarities and differences between (i) and (ii) will become apparent.

Anticipating the need to go beyond isotropic shells we consider cylindrically anisotropic inhomogeneous materials [49] with, in general, four radially varying elastic moduli. Our method of solution uses the formulation of [50] (Note misprints: There is a −**I** on the r.h.s. of eqn (2.8); the left off-diagonal blocks mentioned below (2.16) are positive semi-definite; (*R*_1_, *R*_2_) appearing below eqns (4.1) and (4.4) must be replaced by (*R*_2_, *R*_1_).) although we note that other equivalent state-space approaches have been successfully employed, e.g. Tsukrov & Drach [51] derived displacement and stress solutions for a multilayered composite cylinder with cylindrically orthotropic layers subject to homogeneous boundary loadings using the state space formalism of [52,53]. Our solution method is based on impedance matrices [50] which do not require pointwise solutions for displacement and stress, which simplifies the analysis considerably.

### (a) Matricant and impedance matrices

Given an arbitrary static loading in the far-field, displacement solutions may be written in terms of summations over azimuthal modal dependence of the form e^in*θ*^ for integer *n*. Cylindrical coordinates *r*, *θ* are used here. Radially dependent displacements are then *u*_*r*_(*r*), $u_{\theta }(r)$ with associated traction components *t*_*r*_(*r*) ( = *σ*_*rr*_) and $t_{\theta }(r)\;(=\sigma_{r\theta })$. Assume that the coating (cloak) is cylindrically anisotropic [50] with local orthotropic in-plane anisotropy defined by the moduli (in Voigt notation) *C*_11_, *C*_22_, *C*_12_, *C*_66_, where 1, 2 ↔ *r*, *θ*. The static elastic equilibrium and constitutive equations can then be written as a system of four ordinary differential equations in *r*,

3.1 $$\begin{array}{c} \frac{d\text{v}}{dr}(r)=\frac{1}{r}\text{G}(r)\text{v}(r),\;\text{where} \end{array}$$

3.2 $$\begin{array}{c} \text{v}=\left(\begin{array}{c} u_{r}\\ -iu_{\theta }\\ rt_{r}\\ -irt_{\theta } \end{array}\right),\;\text{G}=\left(\begin{array}{cccc} 1-\gamma & n(\gamma -1) & C_{11}^{-1} & 0\\ -n & 1 & 0 & C_{66}^{-1}\\ C & -nC & \gamma -1 & n\\ -nC & n^{2}C & n(1-\gamma ) & -1 \end{array}\right),\;\begin{array}{rl} \gamma & =1+\frac{C_{12}}{C_{11}},\\ C & =C_{22}-\frac{C_{12}^{2}}{C_{11}}. \end{array} \end{array}$$

The constraint of positive definite strain energy for the two-dimensional deformation requires *C*_11_ > 0, *C*_66_ > 0, *C* > 0.

The propagator, or matricant, **M**(*r*, *r*_0_), by definition [50] satisfies

3.3 $$\frac{d\text{M}}{dr}=\frac{1}{r}\text{G}(r)\text{M}\;\text{with}\;\text{M}(r_{0},r_{0})=\text{I},$$

where **I** is the 4 × 4 identity. Note its important property that

3.4 $$\text{v}(r_{1})=\text{M}(r_{1},r_{0})\text{v}(r_{0})$$

The 2×2 impedance matrix, **Z** (*r*), is defined by

3.5 $$\left(\begin{array}{c} rt_{r}\\ -irt_{\theta } \end{array}\right)=\text{Z}\left(\begin{array}{c} u_{r}\\ -iu_{\theta } \end{array}\right).$$

It can be expressed in terms of the impedance at *r* = *r*_0_, **Z**(*r*_0_), using the matricant, as

3.6 $$\text{Z}(r)=({\text{M}}_{3}+{\text{M}}_{4}\text{Z}(r_{0}))({\text{M}}_{1}+{\text{M}}_{2}\text{Z}(r_{0}){)}^{-1}\;\text{where}\;\text{M}(r,r_{0})=\left(\begin{array}{cc} {\text{M}}_{1} & {\text{M}}_{2}\\ {\text{M}}_{3} & {\text{M}}_{4} \end{array}\right).$$

Alternatively, the impedance satisfies a separate ordinary differential matrix Riccati equation

3.7 $$\begin{array}{rl} & r\frac{d\text{Z}}{dr}+\text{Z}{\text{G}}_{1}+{\text{G}}_{1}^{\text{T}}\text{Z}+\text{Z}{\text{G}}_{2}\text{Z}-{\text{G}}_{3}=0,\\ \text{where}\; & {\text{G}}_{1}=\left(\begin{array}{cc} 1-\gamma & n(\gamma -1)\\ -n & 1 \end{array}\right),\;{\text{G}}_{2}=\left(\begin{array}{cc} C_{11}^{-1} & 0\\ 0 & C_{66}^{-1} \end{array}\right),\;{\text{G}}_{3}=C\left(\begin{array}{cc} 1 & -n\\ -n & n^{2} \end{array}\right). \end{array}$$

The transpose **Z**^T^ satisfies the same equation, and therefore, if the initial condition for the impedance matrix is symmetric then it remains symmetric. We will only consider this case, and can therefore assume that it is always symmetric, **Z**(*r*) = **Z**^T^(*r*). The impedance matrix considered here is the static limit of the dynamic impedance discussed in [54] for general cylindrical anisotropy, specifically the impedance **z** of [54] is related to the present version by $\text{z}=-\text{J}\text{Z}{\text{J}}^{†}$ where J=diag⁡(1,i) and † denotes the Hermitian transpose. Integration of the Riccati equation for the time harmonic problem can be tricky because of the appearance of dynamic resonances, although these difficulties can be circumvented [55]. No such problems arise in the present case, for which numerical integration of (3.7) is stable. The initial value of the impedance for a uniform cylinder is analysed in detail in [54], where it is termed the *central impedance* since the pointwise value of the impedance at *r* = 0 is required for the initial condition of the dynamic Riccati differential equation.

The eigenvalues of **G** are taken to be {*λ*_1_, *λ*_2_, *λ*_3_, *λ*_4_} with right and left eigenvectors **v**_*i*_, **u**_*i*_ (*i* = 1, 2, 3, 4) satisfying **Gv**_*i*_ = **v**_*i*_*λ*_*i*_, ${\text{u}}_{i}^{\text{T}}\text{G}=\lambda_{i}{\text{u}}_{i}^{\text{T}}$ where **V** = [**v**_1_, **v**_2_, **v**_3_, **v**_4_], **U** = [**u**_1_, **u**_2_, **u**_3_, **u**_4_]. The eigenvectors are normalized such that

3.8 $$\begin{array}{rl} & {\text{U}}^{\text{T}}\text{V}=\text{V}{\text{U}}^{\text{T}}=\text{I},\\ & \text{G}=\text{V}\text{D}{\text{U}}^{\text{T}}\Rightarrow {\text{G}}^{m}=\text{V}{\text{D}}^{m}{\text{U}}^{\text{T}},\\ \text{where}\;\; & \text{D}=\text{diag}(\lambda_{1},\;\lambda_{2},\;\lambda_{3},\;\lambda_{4}). \end{array}$$

The eigenvalues and eigenvectors are functions of *r* if the moduli, through **G** depend on *r*.

#### (i) (Uniform properties)

For a constant set of moduli **G** over some range including *r* and *r*_0_, the eigenvalues and eigenvectors are fixed and the solution of (3.3) can be written

3.9 $$\text{M}(r,r_{0})=\text{V}\text{E}(r,r_{0}){\text{U}}^{\text{T}}\;\text{where}\;\text{E}(r,r_{0})=\text{diag}\left({\left(\frac{r}{r_{0}}\right)}^{\lambda_{1}},{\left(\frac{r}{r_{0}}\right)}^{\lambda_{2}},\;{\left(\frac{r}{r_{0}}\right)}^{\lambda_{3}},\;{\left(\frac{r}{r_{0}}\right)}^{\lambda_{4}}\right).$$

Alternatively, **M** can be expressed simply as a matrix exponential,

3.10 $$\text{M}(r,r_{0})=e^{\text{G}\log(r/r_{0})}=(\frac{r}{r_{0}}{)}^{\text{G}}.$$

There are two distinct types of impedance matrix solutions for a uniform medium. The first is the impedance of a solid cylindrical region of finite radius. Since there is no length scale in the impedance relation, it follows that the impedance is independent of the radius, and thus by virtue of equation (3.7), is a solution of the Riccati matrix equation

3.11 $$\text{Z}{\text{G}}_{2}\text{Z}+\text{Z}{\text{G}}_{1}+{\text{G}}_{1}^{\text{T}}\text{Z}-{\text{G}}_{3}=0.$$

The second type of impedance is associated with the dual configuration of an infinite medium with a circular hole of finite radius. Again, the impedance is a root of (3.11). These matrix roots of the algebraic Riccati equation can be found using standard matrix algorithms [56,57].

### (b) Long wavelength scattering

The exterior medium is strictly transversely isotropic but we are interested in planar wave propagation. Hence this two-dimensional problem is isotropic with mechanical behaviour characterized by the two elastic properties *μ*_*e*_ and *K*_*e*_ = *λ*_*e*_ + *μ*_*e*_. The displacement can thus be expressed using two potential functions *ϕ* and *ψ*,

3.12 $$\text{u}=\nabla \phi -\nabla \times \psi {\text{e}}_{3}.$$

Assuming time dependence ${\text{e}}^{-i\omega t}$, the incident wave is in the *x*_1_-direction $\phi =A_{L}e^{ik_{L}x_{1}}$, $\psi =A_{T}e^{ik_{T}x_{1}}$, where *k*_*L*_ = *ω*/*c*_*L*_, *k*_*T*_ = *ω*/*c*_*T*_, $c_{L}^{2}=\left(K_{e}+\mu_{e}\right)/\rho$, $c_{T}^{2}=\mu_{e}/\rho$ and *A*_*L*_ and *A*_*T*_ are the longitudinal and transverse wave amplitudes, respectively. Taking $A_{L}={\left(ik_{L}\right)}^{-1}$, $A_{T}={\left(ik_{T}\right)}^{-1}$ leads to the incident wave

3.13 $$\text{u}=(e^{ik_{L}x_{1}},\;e^{ik_{T}x_{1}},\;0),\;\text{v}={\text{v}}_{L}+{\text{v}}_{T},$$

where, using *x*_1_ = *r*cos*θ*, *x*_2_ = *r*sin*θ*,

3.14 $${\text{v}}_{L}=\left(\begin{array}{c} \cos\theta \\ i\sin\theta \\ ik_{L}r(\lambda_{e}+2\mu_{e}\cos^{2}\theta )\\ -k_{L}r2\mu_{e}\cos\theta \sin\theta \end{array}\right){\text{e}}^{ik_{L}r\cos\theta },\;{\text{v}}_{T}=\left(\begin{array}{c} \sin\theta \\ -i\cos\theta \\ ik_{T}r2\mu_{e}\cos\theta \sin\theta \\ k_{T}r\mu_{e}(\cos^{2}\theta -\sin^{2}\theta ) \end{array}\right){\text{e}}^{ik_{T}r\cos\theta }.$$

In the low-frequency, or equivalently long-wavelength regime, and in the vicinity of the cylinder

3.15 $$k_{L}r\ll 1\;\text{and}\;k_{T}r\ll 1,$$

resulting in the asymptotic expansions

3.16a $$\begin{array}{rl} {\text{v}}_{L} & =\frac{i}{2}k_{L}r\left(\begin{array}{c} 1\\ 0\\ 2(\lambda_{e}+\mu_{e})\\ 0 \end{array}\right)+\left(\begin{array}{c} \cos\theta \\ i\sin\theta \\ 0\\ 0 \end{array}\right)+\frac{i}{2}k_{L}r\left(\begin{array}{c} \cos2\theta \\ i\sin2\theta \\ 2\mu_{e}\cos2\theta \\ i2\mu_{e}\sin2\theta \end{array}\right)+\text{O}(k_{L}^{2}r^{2}), \end{array}$$

3.16b $$\begin{array}{rl} {\text{v}}_{T} & =\frac{1}{2}k_{T}r\left(\begin{array}{c} 0\\ 1\\ 0\\ 0 \end{array}\right)+\left(\begin{array}{c} \sin\theta \\ -i\cos\theta \\ 0\\ 0 \end{array}\right)+\frac{i}{2}k_{T}r\left(\begin{array}{c} \sin2\theta \\ -i\cos2\theta \\ 2\mu_{e}\sin2\theta \\ -i2\mu_{e}\cos2\theta \end{array}\right)+\text{O}(k_{T}^{2}r^{2}). \end{array}$$

These can be considered as *near-field* expansions, valid in the neighbourhood for which (3.15) holds. The first term in **v**_*T*_ is a rigid body rotation, and the second terms in both **v**_*L*_ and **v**_*T*_ are rigid body translations. The first term in **v**_*L*_ can be interpreted as a radially symmetric far-field loading, while the third terms in both **v**_*L*_ and **v**_*T*_ are *n* = ±2 shear-type loadings. The *n* = 1 loadings cause the inclusion to undergo rigid body motion; the parameter that is relevant in the low-frequency limit is the effective mass, or equivalently its effective density. Therefore, at this level of long-wavelength approximation, the scattering can be evaluated from the solutions for *n* = 0 and *n* = ±2 quasi-static loadings. In order to better identify these terms, we rewrite (3.16) as

3.17a $${\text{v}}_{L}=\frac{i}{2}k_{L}r\left(\begin{array}{c} 1\\ 0\\ 2(\lambda_{e}+\mu_{e})\\ 0 \end{array}\right)+\sum_{n=\pm 1}\frac{{\text{e}}^{in\theta }}{2}\left(\begin{array}{c} {\text{a}}_{\pm }\\ 0\\ 0 \end{array}\right)+\frac{i}{4}k_{L}r\sum_{n=\pm 2}e^{in\theta }\left(\begin{array}{c} {\text{a}}_{\pm }\\ 2\mu_{e}{\text{a}}_{\pm } \end{array}\right)+\text{O}(k_{L}^{2}r^{2})$$

and

3.17b $$i{\text{v}}_{T}=\frac{i}{2}k_{T}r\left(\begin{array}{c} 0\\ 1\\ 0\\ 0 \end{array}\right)+\sum_{n=\pm 1}\frac{{\text{e}}^{in\theta }}{2}\left(\begin{array}{c} \pm {\text{a}}_{\pm }\\ 0\\ 0 \end{array}\right)+\frac{i}{4}k_{T}r\sum_{n=\pm 2}e^{in\theta }\left(\begin{array}{c} \pm {\text{a}}_{\pm }\\ \pm 2\mu_{e}{\text{a}}_{\pm } \end{array}\right)+\text{O}(k_{T}^{2}r^{2}),$$

where ${\text{a}}_{\pm }\equiv (\begin{array}{c} 1\\ \pm 1 \end{array})$. The terms in these equations for the incident plane waves can be identified as separate quasi-static loadings of type *n* = 0, 1, and 2. The *n* = 1 term involves only the effective mass term, which involves the average density. This is decoupled from the elasticity problem and will not be discussed further. For the remainder of the paper, we will focus on the *n* = 0 and *n* = 2 loadings.

## 4. Effective bulk modulus: *n* = 0

Equations (3.1) and (3.2) simplify for *n* = 0 to two uncoupled systems

4.1 $$\frac{d}{dr}\left(\begin{array}{c} u_{r}\\ rt_{r} \end{array}\right)=\frac{1}{r}\left(\begin{array}{cc} 1-\gamma & C_{11}^{-1},\\ C & \gamma -1 \end{array}\right)\left(\begin{array}{c} u_{r}\\ rt_{r} \end{array}\right)$$

and

4.2 $$\frac{d}{dr}\left(\begin{array}{c} u_{\theta }\\ rt_{\theta } \end{array}\right)=\frac{1}{r}\left(\begin{array}{cc} 1 & C_{66}^{-1}\\ 0 & -1 \end{array}\right)\left(\begin{array}{c} u_{\theta }\\ rt_{\theta } \end{array}\right).$$

The latter is associated with pure twist or torsion: define the relationship between the angle of twist and the angular traction as $r^{-1}u_{\theta }=S_{e}(r)t_{\theta }$ then equation (4.2) implies that the effective compliance is

4.3 $$S_{e}(r)=\frac{r^{2}}{r_{0}^{2}}S_{0}+r^{2}\int_{r_{0}}^{r}\frac{dx}{x^{3}C_{66}(x)},$$

where *S*_0_ = *S*_*e*_(*r*_0_). For instance, *S*_0_ = 0 for a shell *r* > *r*_0_ pinned at *r* = *r*_0_.

Our main concern with the *n* = 0 case is for radially symmetric loading for which the only quantity of importance is the effective compressibility of the inclusion. Define the pointwise effective bulk modulus $K_{*}$ as a function of *r*, by

4.4 $$K_{*}(r)\equiv \frac{rt_{r}}{2u_{r}}.$$

Matching this to the exterior medium guarantees an NI effect for *n* = 0, in addition to zero monopole scattering in the low-frequency regime. We next derive $K_{*}(r)$.

### (a) A scalar Riccati equation for the bulk modulus

Substituting $rt_{r}=2K_{*}u_{r}$ in (4.1) yields the Riccati ordinary differential equation

4.5 $$\frac{dK_{*}}{dr}+\frac{2}{r}C_{11}^{-1}(K_{*}^{2}-C_{12}K_{*}-\frac{1}{4}(C_{11}C_{22}-C_{12}^{2}))=0.$$

Noting that $C_{11}C_{22}-C_{12}^{2}>0$, define the positive moduli *K*, *μ* and the non-dimensional parameter *β* > 0

4.6 $$K=\frac{1}{2}(\sqrt{C_{11}C_{22}}+C_{12}),\;\mu =\frac{1}{2}(\sqrt{C_{11}C_{22}}-C_{12}),\;\beta =\sqrt{\frac{C_{22}}{C_{11}}},$$

so that the Riccati equation becomes

4.7 $$\frac{dK_{*}}{dr}+\frac{2}{r}\beta {\left(K+\mu \right)}^{-1}\;(K_{*}-K)(K_{*}+\mu )=0.$$

### (b) Example: Constant moduli

If *K*, *μ* and *β* are constants the Riccati equation (4.7) can be integrated and combined with the matching conditions at the core boundary, $K_{*}(r_{0})=K_{0}$, and at the exterior boundary, $K_{e}=K_{*}(r_{1})$, *r*_1_ ≥ *r*_0_, to yield

4.8 $$\frac{K-K_{e}}{\mu +K_{e}}=f^{\beta }\;\left(\frac{K-K_{0}}{\mu +K_{0}}\right)\;\text{where}\;f=\frac{r_{0}^{2}}{r_{1}^{2}}.$$

This is in agreement with (2.1b) when *β* = 1, but is more general in that it includes the possibility of an anisotropic layer (*β* ≠ 1). For given values of the inner and outer parameters *K*_0_, *K*_*e*_ and radii *r*_0_, *r*_1_, the relation (4.8) places a constraint on the possible cloaking moduli. In this case, it relates *K*, *μ* and *β* according to

4.9 $$\mu =-\left(\frac{K_{0}(K_{e}-K)-K_{e}(K_{0}-K)f^{\beta }}{(K_{e}-K)-(K_{0}-K)f^{\beta }}\right).$$

For instance, taking *K*_0_ → ∞, 0, implies the limiting cases

4.10 $$\mu =\left\{ \begin{array}{ll} (K_{e}-K)f^{-\beta }-K_{e}, & \text{rigid core},\\ ((K_{e}^{-1}-K^{-1})f^{-\beta }-K_{e}^{-1}{)}^{-1}, & \text{hole}. \end{array}\right.$$

At this stage, there are still two unknowns, *K* and *μ*, and only one relation between them, equation (4.9). Choosing coatings with in-plane shear and bulk moduli and anisotropy ratio *β* that satisfy the relationship (4.9) thus ensures an NI when the medium is subjected to in-plane hydrostatic pressure. In order to find a second relationship between *μ* and *K* (and thus uniquely define the coating properties), a second relation needs to be determined, if one exists. It transpires that this second relationship comes from the *n* = 2 solution.

## 5. Effective shear modulus: *n* = 2

We first consider the cloaking layer to be isotropic, and prove that it is not possible to obtain a strong NI (static cloak). We will then show that the strong NI condition can only be met with an anisotropic layer.

### (a) Isotropic medium

An isotropic shell has two elastic parameters which can be taken as *C*_66_ and *γ*, in terms of which the remaining two elastic moduli in equation (3.2) are *C*_11_ = 2 *C*_66_(2 − *γ*)^−1^ and *C* = 2 *C*_66_*γ*. The eigenvalues of **G** are *n* − 1, *n* + 1, 1 − *n*, −1 − *n*, which for *n* = 2 become {*λ*_1_, *λ*_2_, *λ*_3_, *λ*_4_} = {1, 3, − 1, − 3}. The right and left eigenvectors satisfying (3.8) are

5.1 U=12C[2γ−310    −γ31−3γ    2−1−12γ−2    2−γ1−12+γ],V=12C−1[12γ−23213    12+γ32−γ−13    10−2γ−1    1γγ1],

where **C** = diag(2*C*_66_,  2*C*_66_,  1,  1), $\gamma =\frac{1}{1-\nu }$ and *ν* is Poisson’s ratio.

Consider a solid cylinder. Only solutions with *λ*_*i*_ ≥ 0 are permissible in the cylinder, corresponding to the first two columns of **V** in (5.1). The impedance matrix at every point in the cylinder is then constant and equal to

5.2 $$\text{Z}={\text{V}}_{3}{\text{V}}_{1}^{-1}=\frac{2C_{66}}{4-\gamma }\left(\begin{array}{cc} 2+\gamma & 2-2\gamma \\ 2-2\gamma & 2+\gamma \end{array}\right)\;\text{where}\;\text{V}=\left(\begin{array}{cc} {\text{V}}_{1} & {\text{V}}_{2}\\ {\text{V}}_{3} & {\text{V}}_{4} \end{array}\right),$$

in agreement with [54, eqn (8.6)] for the central impedance matrix. It may be checked that **Z** of (5.2) solves the Riccati equation (3.11).

### (b) Neutral inclusion shear condition

A cylinder of uniform material with shear modulus and Poisson’s ratio *μ*_0_, *ν*_0_ and radius *r*_0_ is surrounded by a shell, or cloak with outer radius *r*_1_. The impedance on the exterior boundary is, see (3.6)

5.3 $$\text{Z}(r_{1})=({\text{M}}_{3}+{\text{M}}_{4}\text{Z}(r_{0}))({\text{M}}_{1}+{\text{M}}_{2}\text{Z}(r_{0}){)}^{-1},\;\text{Z}(r_{0})=\frac{2\mu_{0}}{3-4\nu_{0}}\left(\begin{array}{cc} 3-2\nu_{0} & -2\nu_{0}\\ -2\nu_{0} & 3-2\nu_{0} \end{array}\right),$$

and **M**_*i*_ are block elements of the matricant **M** (*r*_1_, *r*_0_). The far-field loading for *n* = 2 (*n* = −2 is different!) follows from (3.17). In addition, the exterior field in *r* > *r*_1_ comprises the solutions with *λ*_*i*_ < 0 which are **v**_3_ and **v**_4_, the third and fourth columns in **V** of (5.1). The continuity condition at the interface *r* = *r*_1_ for some incident amplitude *α*_1_ ≠ 0 is

5.4 $$\alpha_{1}\left(\begin{array}{c} 1\\ 1\\ 2\mu_{e}\\ 2\mu_{e} \end{array}\right)+\alpha_{3}{\text{v}}_{3}+\alpha_{4}{\text{v}}_{4}=\left(\begin{array}{c} \text{b}\\ \text{Z}(r_{1})\text{b} \end{array}\right),$$

where *μ*_*e*_ is the exterior shear modulus and $\text{b}{\left(u_{r}(r_{1}),-\text{i}u_{\theta }(r_{1})\right)}^{T}$. The strong NI condition requires that *α*_3_ = 0, *α*_4_ = 0, in which case we have

5.5 $$\text{b}=\alpha_{1}\left(\begin{array}{c} 1\\ 1 \end{array}\right),\;\text{Z}(r_{1})\text{b}=2\mu_{e}\alpha_{1}\left(\begin{array}{c} 1\\ 1 \end{array}\right)\;\text{for a strong NI}.$$

Hence, the strong NI condition is that (1 1)^T^ is an eigenvector of **Z**(*r*_1_) with eigenvalue 2*μ*_*e*_. The first of these requires that

5.6 $$Z_{11}+Z_{12}=Z_{21}+Z_{22},$$

which can be simplified using the fact that the impedance is symmetric, *Z*_12_ = *Z*_21_. Thus, the cylindrical region *r* ≤ *r*_1_ acts as a strong NI if and only if the elements of the impedance matrix satisfy

5.7 $$Z_{11}(r_{1})=Z_{22}(r_{1})\;\text{for a strong NI}.$$

#### (i) (Isotropic core plus shell)

Consider a core *μ*_0_, *ν*_0_ of radius *r*_0_ with a surrounding shell *μ*, *ν* of outer radius *r*_1_ > *r*_0_. Using equations (3.9), (5.1) and (5.3), it can be shown that

5.8 $$Z_{11}(r_{1})-Z_{22}(r_{1})=3\left(\frac{r_{1}^{2}}{r_{0}^{2}}-1\right)\frac{\left(\mu_{0}-\mu \right)}{(1-\nu )\mu }(\mu +\frac{\mu_{0}}{3-4\nu_{0}})/\det({\text{M}}_{1}(r_{1},r_{0})+{\text{M}}_{2}(r_{1},r_{0})\text{Z}(r_{0})).$$

The NI condition (5.7) can only be met if the shell and core have the same shear modulus, *μ*_1_ = *μ*_0_, in which case the effective shear modulus is simply *μ*_0_, regardless of the values of Poisson’s ratios *ν*_1_ and *ν*_0_, see equation (5.21). This means that the core cannot be transformed into a strong NI by surrounding it with a single shell of isotropic material.

### (c) Low-frequency transparency condition in shear

For a given incident wave, the *n* = 2 contribution to the scattered displacement **u**^*s*^ exterior to the inclusion can be expressed using equation (3.12) with $\phi =B_{L}H_{2}^{\left(1\right)}(k_{L}r){\text{e}}^{i2\theta }$, $\psi =iB_{T}H_{2}^{\left(1\right)}(k_{T}r){\text{e}}^{i2\theta }$, where $H_{n}^{\left(1\right)}$ is the Hankel function of the first kind. This yields, dropping the e^i2*θ*^ term,

5.9 $$\begin{array}{rl} u_{r}^{s} & =k_{L}B_{L}H_{2}^{{\left(1\right)}^{\prime }}(k_{L}r)+2\frac{B_{T}}{r}H_{2}^{\left(1\right)}(k_{T}r),\\ \text{and}\;\;-iu_{\theta }^{s} & =k_{T}B_{T}H_{2}^{{\left(1\right)}^{\prime }}(k_{T}r)+2\frac{B_{L}}{r}H_{2}^{\left(1\right)}(k_{L}r). \end{array}\}$$

Both *B*_*L*_ and *B*_*T*_ are functions of frequency, the precise forms dependent on the inclusion details. For the moment, we assume that they each have regular expansions about *ω* = 0, i.e.

5.10 $$\begin{array}{rl} & B_{L}=B_{L0}+\omega B_{L1}+\ldots ,\\ \text{and}\;\; & B_{T}=B_{T0}+\omega B_{T1}+\ldots . \end{array}\}$$

Expanding (5.9) in the long wavelength near-field limit, the scattered wave is to leading order in *ω*,

5.11 $$\left(\begin{array}{c} u_{r}^{s}\\ iu_{\theta }^{s} \end{array}\right)=-\frac{2i}{\pi r}\left(\begin{array}{c} B_{T0}\\ B_{L0} \end{array}\right)+\frac{8i}{\pi r^{3}}(\frac{B_{L0}}{k_{L}^{2}}-\frac{B_{T0}}{k_{T}^{2}})\left(\begin{array}{c} 1\\ -1 \end{array}\right)+\ldots .$$

This low-frequency expansion should be consistent with the purely static representation of the exterior ‘scattered’ field as a sum of the form, see equation (5.4),

5.12 $${\text{v}}^{s}=\alpha_{3}{\text{v}}_{3}+\alpha_{4}{\text{v}}_{4},$$

where **v**_3_ and **v**_4_ are the third and fourth columns in **V** of (5.1), corresponding to *r*^−1^ and *r*^−3^ decay outside the inclusion, respectively. Comparing the *r*^−1^ term in (5.11) with the first two elements of **v**_3_ implies that

5.13 $$\frac{B_{L0}}{B_{T0}}=\frac{2-\gamma }{2}\Rightarrow \frac{B_{L0}}{k_{L}^{2}}=\frac{B_{T0}}{k_{T}^{2}},$$

because $1-(\gamma /2)=k_{L}^{2}/k_{T}^{2}$. Equation (5.13) means that if one of *B*_*L*0_, *B*_*T*0_ vanishes, then both vanish. Equivalently, it says that both *B*_*L*0_, *B*_*T*0_ vanish if the coefficient of the *r*^−1^ term is zero.

Hence, the leading-order term in the low-frequency expansion of the scattered field vanishes if and only if the coefficient of the *r*^−1^ term, i.e. **v**_3_, in the quasi-static solution is zero. Low-frequency transparency therefore requires only that *α*_3_ vanishes. This result agrees with the strain energy condition first derived by Christensen & Lo [33], and later in more general form by Hashin [34] and Hervé & Zaoui [35]. Also, the above derivation is independent of the type of incident wave, but relies only on the form of the scattered wave potentials as a combination of Hankel functions.

In summary, we conclude that

**Lemma 5.1.**

*Low-frequency transparency in shear, is obtained if* (*see equation (5.12)*)

5.14 $$\alpha_{3}=0,$$

*which is equivalent to the weak NI condition for in-plane deformation, if (4.9) also holds (the bulk modulus condition) and β* = 1 *for isotropic coatings*.

*The core–shell composite is a strong NI for planar deformation if and only if*

5.15 $$\alpha_{3}=0\;\text{and}\;\alpha_{4}=0.$$

*together with (4.9)*.

We next seek more explicit versions of these conditions, and in the process find the effective shear modulus of the matrix.

#### (i) (The effective shear modulus)

Equation (5.4) can be written

5.16 $$\left[-{\text{v}}_{3}\;\;-{\text{v}}_{4}\;\;\begin{array}{c} \text{I}\\ \text{Z}(r_{1}) \end{array}\right]\left(\begin{array}{c} \alpha_{3}\\ \alpha_{4}\\ \text{b} \end{array}\right)=\alpha_{1}\left(\begin{array}{c} 1\\ 1\\ 2\mu_{e}\\ 2\mu_{e} \end{array}\right).$$

The transparency/weak NI condition (5.14) then becomes

5.17 $$\det\left[{\text{v}}_{1}\;\;{\text{v}}_{4}\;\;\begin{array}{c} \text{I}\\ \text{Z}(r_{1}) \end{array}\right]=0\Rightarrow \det\left(\begin{array}{cccc} \frac{1}{2} & \frac{1}{6} & 1 & 0\\ \frac{1}{2} & -\frac{1}{6} & 0 & 1\\ \mu_{e} & -\mu_{e} & Z_{11} & Z_{12}\\ \mu_{e} & \mu_{e} & Z_{12} & Z_{22} \end{array}\right)=0.$$

Expanding the determinant yields a quadratic equation for the effective shear modulus

5.18 $$\mu_{e}^{2}-\frac{\mu_{e}}{6}(Z_{11}+Z_{22}+4Z_{12})-\frac{1}{12}(Z_{11}Z_{22}-Z_{12}^{2})=0.$$

The sign of the root chosen must agree with the NI value for the effective modulus above when condition (5.7) holds.

The above results for both the low-frequency transparency and the weak and strong NI conditions can be combined with the bulk modulus condition of §4 as follows.

**Theorem 5.2.**

*The cylindrical core–shell is transparent at low frequency and acts as a weak NI for in-plane hydrostatic pressure and in-plane shear if the exterior medium has bulk and shear moduli*

5.19a $$K_{e}=\frac{K(\mu +K_{0})-\mu (K-K_{0}){\left(r_{0}/r_{1}\right)}^{2\beta }}{\mu +K_{0}+(K-K_{0}){\left(r_{0}/r_{1}\right)}^{2\beta }}$$

*and*

5.19b $$\mu_{e}=\frac{1}{6}\left(Z_{s}+2Z_{12}+\sqrt{{\left(2Z_{s}+Z_{12}\right)}^{2}-3Z_{d}^{2}}\right),$$

*where*

5.19c $$K=\frac{1}{2}(\sqrt{C_{11}C_{22}}+C_{12}),\;\mu =\frac{1}{2}(\sqrt{C_{11}C_{22}}-C_{12}),\;\beta =\sqrt{\frac{C_{22}}{C_{11}}}$$

*and*

5.19d $$Z_{s}=\frac{1}{2}(Z_{11}+Z_{22}),\;Z_{d}=\frac{1}{2}(Z_{11}-Z_{22}),$$

*and Z*_*ij*_ *are the elements of the impedance matrix* **Z**(*r*_1_) *defined by equation* (5.3).

*Furthermore, the layered shell acts as a strong NI for in-plane shear and hydrostatic loading, if and only if*

5.20 $$Z_{d}=0,$$

*coupled with (5.19a). The strong NI condition (5.20) cannot occur if the shell is isotropic, but requires anisotropy. If the strong NI condition is met then the matrix shear modulus is*

5.21 $$\mu_{e}=\frac{1}{2}(Z_{11}+Z_{12}).$$

*and the matrix bulk modulus is K*_*e*_ *of (5.19a)*.

In practice of course the *core* and *matrix/exterior* properties are specified and the coating properties (and thickness) are deduced by solving (5.21) and (5.19a).

In summary, low-frequency transparency/weak NI can be achieved with isotropic shell material. The strong NI condition restricts the types of shells: equation (5.8) indicates that a uniform isotropic shell cannot yield a strong NI, regardless of the isotropic core properties. We note that the explicit form of *μ*_*e*_ in (5.19b) is far simpler than the alternatives available [34, eqn (50)], [35, eqn (82)], even the original [33, eqn (4.11)]. Finally, it should be kept in mind that, just as for the approximate NI considered in §2, the weak and strong NI conditions are not guaranteed to be achievable for all combinations of matrix and core properties and core volume fraction in the composite cylinder.

## 6. Implementation of the theory: weak versus strong neutral inclusions

We now provide some examples of strong NIs. In particular, for each of the examples in table 1 the identities (5.19a) and (5.20) are satisfied, and hence the composite cylinder is a strong NI.

**Table 1.** Examples of composite cylinder strong NIs. The isotropic core radius and properties are *r*_0_, *μ*_0_, *ν*_0_ and the exterior (matrix) properties are the in-plane bulk and shear moduli *K*_*e*_ and *μ*_*e*_. The anisotropic coating (shell) properties required are *C*_11_, *C*_22_, *C*_12_ and *C*_66_ and its outer radius is *r*_1_ = 1.

example	*r*_0_/*r*_1_	*μ*_0_	*ν*_0_	*C*_11_	*C*_22_	*C*_12_	*C*_66_	*K*_*e*_	*μ*_*e*_
(i)	0.5	1	$\frac{1}{3}$	4.0	4.40	2.49	1.0	3.2572	0.9401
(ii)	0.2	0	$\frac{1}{3}$	4.8	2.9781	2.80	1.0	1.9782	0.6445
(iii)	0.2	10^6^	$\frac{1}{3}$	2.5782	2.52	2.4	1.0	2.5848	0.2990
(iv)	0.75	21	$\frac{1}{3}$	3.5839	11.5096	−0.4658	3.2078	6.0307	5.9049

**Figure 2.**
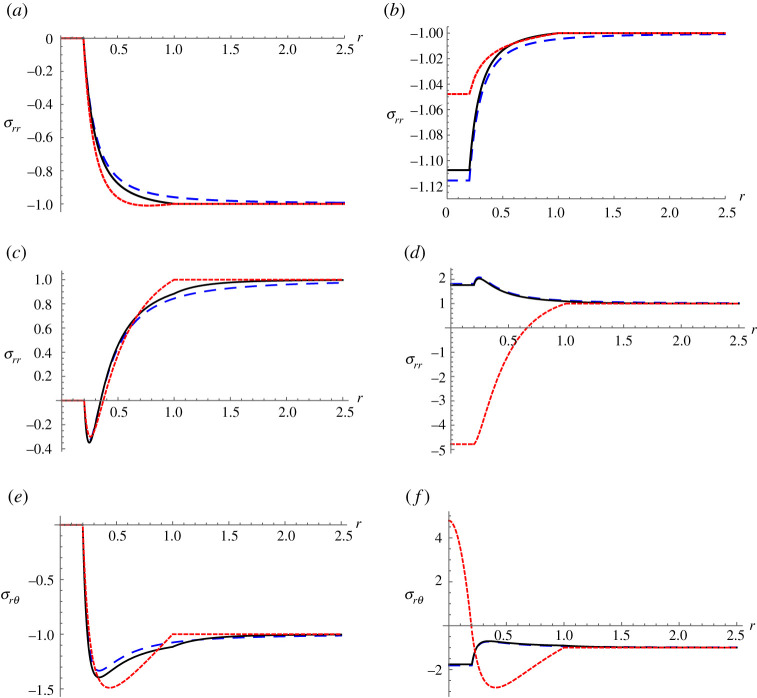
Stress plots as functions of *r*, associated with an isolated fibre (blue long dash), weak (isotropic) NI (black solid), strong (anisotropic) NI (red short dash). (*a*,*c*,*e*): Example (ii) and (*b*,*d*,*e*): Example (iii) from table 1. (*a*) and (*b*) illustrate *σ*_*rr*_(*r*, *θ* = 0) associated with in-plane hydrostatic pressure for Examples (ii) and (iii), respectively, whereas (*c*,*d*) and (*e*,*f* ) illustrate *σ*_*rr*_(*r*, *θ* = 0) and *σ*_*rθ*_(*r*, *θ* = *π*/4) associated with in-plane shear, for Examples (ii) and (iii), respectively. Far field loading is normalized to unity. (Online version in colour.)

In figure 2, we plot radial and shear stress distributions as functions of *r* associated with Example (ii) (left of the figure) and (iii) (right of the figure) in table 1. For all cases, we fix the fibre and exterior (matrix) properties and plot the isolated fibre (no coating) case (blue long dash), the weak NI case (black solid) and the strong NI case (red short dash). Weak NI properties are deduced from the Christensen and Lo shear conditions (see appendix B) coupled with the standard isotropic bulk modulus condition (5.19a) with *β* = 1 (isotropic coating). We thus deduce that for example (ii) isotropic coating properties are $K^{\mathrm{weak}}=2.32912,$ $\mu^{\mathrm{weak}}=0.715023$, whereas for example (iii) isotropic coating properties are $K^{\mathrm{weak}}=2.47029,$ $\mu^{\mathrm{weak}}=0.277903$.

Figure 2*a*,*b* illustrates *σ*_*rr*_(*r*) for the *hydrostatic problem* (independent of *θ*) for Examples (ii) and (iii), respectively. The problem is scaled such that *σ*_*rr*_ → 1 as *r* → ∞. Figure 2*c*–*e* illustrates the *shear problem (*σ*_*xx*_ + *σ*_*yy*_ = 0 as *r* → ∞)*. Figure 2*c*,*e* correspond to Example (ii) whereas figure 2*d*,*f* correspond to Example (iii). *σ*_*rr*_ is evaluated at *θ* = 0 and *σ*_*rθ*_ is evaluated at *θ* = *π*/4.

One should note that strong NIs ensure that the field is unperturbed in *r* ≥ 1 for the shear problem. The hydrostatic problem is unperturbed for *r* ≥ 1 for both strong *and* weak NIs as expected. The effect is more noticeable in Example (ii) (void) than for Example (iii) (rigid core). In the latter case, the weak NI can be seen as almost as effective as the strong NI. In the former, however, the weak NI is ineffective in shear. In Example (iii), we also note that the strong NI has a strong influence on the stress distribution in the core.

## 7. Conclusion

The connection between low-frequency transparency of elastic waves and NIs has been made for the first time. Intuitively, both effects are related to static or quasi-static cloaking, although as we have seen, the relationships require careful definitions of both NIs and low-frequency transparency. Two distinct types of NI have been identified, weak and strong, with the former equivalent to low-frequency transparency and the latter with static cloaking. The main results of the paper are summarized in theorem 5.2 which shows that weak NI/low-frequency transparency is easier to achieve than strong NI/static cloaking. The former can be obtained with an isotropic shell surrounding the core [42], while the latter requires anisotropy in the shell/cloak. For a given core and matrix, and relative shell thickness, the determination of the shell properties for either the weak or strong NI effect is implicit through effective medium conditions. The existence of solutions is not guaranteed, but depends upon the parameters in a non-trivial manner.

The problem has been made tractable by considering the *n* = 0, 1, 2 sub-problems, with *n* = 1 trivially related to density. The concepts of low-frequency wave transparency and NI are identical for the *n* = 0 problem, for which there is no distinction between weak and strong NI effects. Thus, if the exterior bulk modulus matches the effective bulk modulus of the core–shell composite cylinder then the latter acts as an NI and is transparent in the long wavelength regime. Distinguishing between weak and strong NI effects are necessary for the *n* = 2 problem. For the weak NI effect, the shell properties must be such that the single condition (5.17) holds, in which case the effective shear modulus of the shell plus core is given by (5.19b). The strong NI effect requires that equations (5.17) and (5.20) are both satisfied, with matrix effective shear modulus of equation (5.21).

These connections between low-frequency transparency, static cloaking and NIs provide the material designer with options for achieving elastic cloaking in the quasi-static limit. Extension of the results to spherical geometries is the natural next step and will be the subject of a future report.

## Data accessibility

This work does not have any experimental data.

## Appendix A. Elastodynamic scattering solution

Based on the representation (3.12), let

A 1 $$\begin{array}{rl} & \phi =(B_{L}J_{n}(k_{L}r)+D_{L}H_{n}^{\left(1\right)}(k_{L}r))k_{L}^{-1}e^{in\theta }\\ \text{and}\; & \psi =(B_{T}J_{n}(k_{T}r)+D_{T}H_{n}^{\left(1\right)}(k_{T}r))k_{T}^{-1}e^{in\theta }, \end{array}\}$$

then, dropping the $e^{in\theta }$ terms,

A 2a $$\begin{array}{rl} & \text{u}={\text{U}}_{n}(J_{n},r)\text{b}+{\text{U}}_{n}(H_{n}^{\left(1\right)},r)\text{d}, \end{array}$$

A 2b $$\begin{array}{rl} & r\text{t}={\text{V}}_{n}(J_{n},r)\text{b}+{\text{V}}_{n}(H_{n}^{\left(1\right)},r)\text{d}\;\text{where} \end{array}$$

A 2c $$\begin{array}{rl} & \text{u}=\left(\begin{array}{c} u_{r}\\ u_{\theta } \end{array}\right),\;\text{t}=\left(\begin{array}{c} t_{r}\\ t_{\theta } \end{array}\right),\;\text{b}=\left(\begin{array}{c} B_{L}\\ B_{T} \end{array}\right),\;\text{d}=\left(\begin{array}{c} D_{L}\\ D_{T} \end{array}\right), \end{array}$$

A 2d $$\begin{array}{rl} & {\text{U}}_{n}(f,r)=\left(\begin{array}{cc} f^{\prime }(k_{L}r) & -\frac{in}{k_{T}r}f(k_{T}r)\\ \frac{in}{k_{L}r}f(k_{L}r) & f^{\prime }(k_{T}r) \end{array}\right), \end{array}$$

A 2e $$\begin{array}{rl} \text{and}\; & {\text{V}}_{n}(f,r)=\mu_{e}\left(\begin{array}{cc} k_{L}r[2f^{\prime \prime }(k_{L}r)+(2-\frac{k_{T}^{2}}{k_{L}^{2}})f(k_{L}r)] & -2in[f^{\prime }(k_{T}r)-\frac{1}{k_{T}r}f(k_{T}r)]\\ 2in[f^{\prime }(k_{L}r)-\frac{1}{k_{L}r}f(k_{L}r)] & -2f^{\prime }(k_{T}r)+(\frac{2n^{2}}{k_{T}r}-k_{T}r)f(k_{T}r) \end{array}\right). \end{array}$$

Following the notation of [54], assume

A 3 $$r\text{t}=-{\text{Z}}_{1}\text{u}\;\text{at}\;r=r_{1},$$

then the scattered L and T amplitudes **d** of azimuthal order *n* can be found in terms of the incident ones **b** as

A 4 $$\text{d}=-({\text{V}}_{n}(H_{n}^{\left(1\right)},r_{1})+{\text{Z}}_{1}{\text{U}}_{n}(H_{n}^{\left(1\right)},r_{1}){)}^{-1}({\text{V}}_{n}(J_{n},r_{1})+{\text{Z}}_{1}{\text{U}}_{n}(J_{n},r_{1}))\text{b}.$$

This is the basic equation for solving the scattering.

The impedance **Z**_1_ is found by first forming the core impedance, which follows from [54, eqn (8.9)]. This serves as the initial condition for integrating the impedance from *r* = *r*_0_ to *r*_1_. Direct integration of the dynamic analogue of the Riccati equation (3.7) is unstable, however, fast and stable methods exist to circumvent this difficulty. We use the Möbius scheme based on eqns (17) and (32) of [55].

## Appendix B. Christensen and Lo’s weak neutral inclusion

Christensen & Lo [33,47] developed the so-called Generalized Self-Consistent scheme and thus provided the following expressions for the effective (exterior) shear modulus *μ*_*e*_ in terms of the core *μ*_0_ and shell *μ* properties. We have re-written these here since we specify core and exterior properties and solve for coating properties. We have also corrected the typographical errors that appeared in the original paper:

B 1 $$D{\left(\frac{\mu }{\mu_{e}}\right)}^{2}+B\frac{\mu }{\mu_{e}}+A=0,$$

where

B 2 $$\begin{array}{rl} D & =3f{\left(1-f\right)}^{2}\left(\frac{\mu_{0}}{\mu }-1\right)\left(\frac{\mu_{0}}{\mu }+\eta_{0}\right)\\ \; & +\left(\frac{\mu_{0}}{\mu }\eta +\left(\frac{\mu_{0}}{\mu }-1\right)f+1\right)\left(\frac{\mu_{0}}{\mu }+\eta_{0}+\left(\left(\frac{\mu_{0}}{\mu }\right)\eta -\eta_{0}\right)f^{3}\right), \end{array}$$

B 3 $$\begin{array}{rl} B & =-6f{\left(1-f\right)}^{2}\left(\frac{\mu_{0}}{\mu }-1\right)\left(\frac{\mu_{0}}{\mu }+\eta_{0}\right)\\ & \;+\left(\frac{\mu_{0}}{\mu }\eta +\left(\frac{\mu_{0}}{\mu }-1\right)f+1\right)\left((\eta -1)\left(\frac{\mu_{0}}{\mu }+\eta_{0}\right)-2\left(\left(\frac{\mu_{0}}{\mu }\right)\eta -\eta_{0}\right)f^{3}\right),\\ & \;+(\eta +1)f\left(\frac{\mu_{0}}{\mu }-1\right)\left(\frac{\mu_{0}}{\mu }+\eta_{0}+\left(\frac{\mu_{0}}{\mu }-\eta_{0}\right)f^{3}\right) \end{array}$$

B 4 $$\begin{array}{rl} \mathrm{and}\;\; & A=3f{\left(1-f\right)}^{2}\left(\frac{\mu_{0}}{\mu }-1\right)\left(\frac{\mu_{0}}{\mu }+\eta_{0}\right)\\ & \;+\left(\frac{\mu_{0}}{\mu }\eta +\eta_{0}\eta +\left(\frac{\mu_{0}}{\mu }-\eta_{0}\right)f^{3}\right)\left(\left(\frac{\mu_{0}}{\mu }-1\right)\eta -\left(\frac{\mu_{0}}{\mu }\eta +1\right)\right), \end{array}$$

with $f=r_{0}^{2}/r_{1}^{2}$, *η* = 1 + 2(*μ*/*K*) and *η*_0_ = 1 + 2(*μ*_0_/*K*_0_).
